# Investigation of a DNA Profiling Method Using Only Cementum More Than 70 Years After Death

**DOI:** 10.7759/cureus.56998

**Published:** 2024-03-26

**Authors:** Yuna Miura, Masatsugu Hashimoto, Yasutaka Nakamura, Noboru Ishikawa

**Affiliations:** 1 Department of Forensic Odontology and Anthropology, Tokyo Dental College, Chiyoda-ku, JPN

**Keywords:** tooth, personal identification, str profiling, cementum, forensic odontology

## Abstract

Short tandem repeat (STR) typing is widely used not only for blood relationship identification but also for the personal identification of unidentified bodies. However, DNA is susceptible to the effects of environmental factors, consequently leading to reduced DNA yields. Therefore, to maximize the DNA yield required for identification, teeth are generally completely pulverized during DNA extraction. However, this renders subsequent testing after DNA profiling impossible. In this study, we investigated the utility of DNA profiling using only the cementum from teeth that had been left outdoors for long postmortem intervals. We analyzed 90 molars (fresh teeth) that were extracted within six months at a dental clinic and 90 molars (stale teeth) exposed outdoors for over 70 years, and following cementum extraction, the accuracy of STR profiling, optimal site for cementum collection, and minimum amount of cementum required for STR profiling were determined. The results demonstrated that the profiling accuracy of DNA extracted from cementum was comparable to that of DNA from dental pulp and dentin. Furthermore, the collection of cementum from either near the cervical line or from the root apex areas did not show significant differences in DNA profiling accuracy, indicating that securing at least 5 mg of cementum was sufficient to ensure precise DNA profiling. These findings suggest that DNA profiling using only cementum is viable even in teeth that have been subjected to a long postmortem interval.

## Introduction

Teeth serve as a valuable source of information for personal identification owing to their durability, distinctiveness, and recordability. In particular, because of their high degree of preservation, it is possible to use dental records not only for personal identification but also for estimating race, age, and DNA profiling [1,2]. Personal identification using DNA profiling is highly discriminatory and is typically used for identifying severely decomposed or skeletonized human remains [3-5]. However, DNA is highly susceptible to degradation because of the storage environment of the samples (owing to factors such as temperature, humidity, and pH), which results in a reduced DNA yield [6-9]. To maximize the DNA yield, teeth are typically pulverized completely, and DNA is primarily extracted from the dental pulp and cementum to obtain the necessary DNA yield for DNA profiling [10]. However, if the samples are completely pulverized, it is impossible to conduct any subsequent testing after DNA profiling. Recent studies have shown that only cementum can provide a DNA concentration sufficient for short tandem repeat (STR) profiling, and it has been reported that STR profiling is possible even with teeth recovered from bodies less than three years postmortem [11-16]. The cementum is a hard tissue that covers tooth roots and anchors the teeth to the alveolar bone through the insertion of Sharpey’s fibers from the periodontal ligament. Furthermore, cementum is classified into cellular and acellular cementum, and it has been suggested that the DNA necessary for identification can be obtained from cementocytes abundantly present in the root apex and root bifurcation areas [17]. However, no studies have investigated the feasibility of conducting STR profiling using only cementum from teeth retrieved from bodies that have undergone an extended postmortem interval or from teeth that are in a state of poor preservation. In this study, we tested the hypothesis that STR profiling using only cementum would be possible even when using teeth that had undergone an extended postmortem interval.

## Materials and methods

Sample preparation

**Figure 1:**
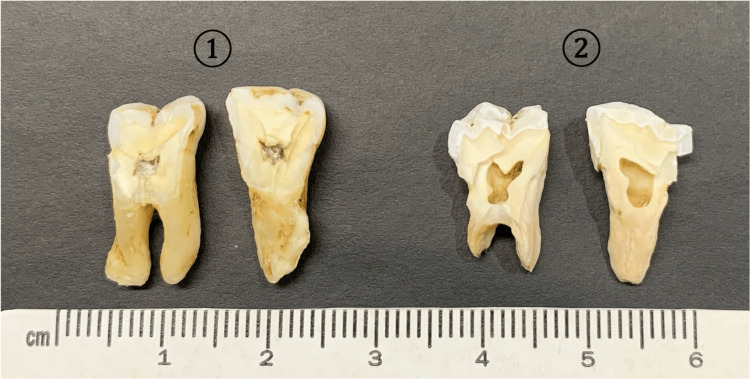
The cut sections of the fresh tooth ① and stale tooth ② Fresh teeth were those that had been extracted within six months, and stale teeth were those that had been left outdoors for more than 70 years.

This study included 90 molars (fresh teeth) that were extracted within six months at Tokyo Dental College, with patient consent, along with 90 molars (stale teeth) recovered from corpses that had been buried outdoors in the same area for over 70 years (Figure 1).

**Figure 2:**
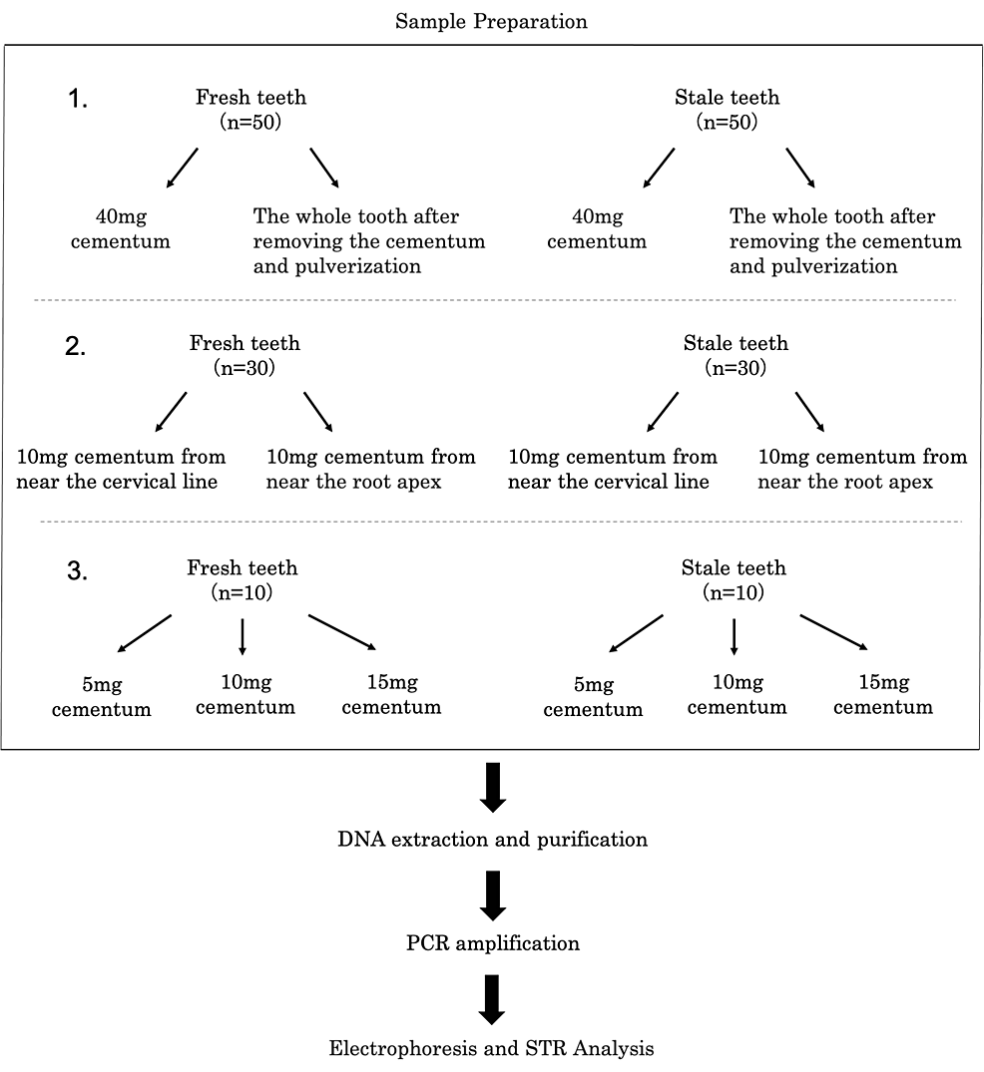
Flowchart from sample preparation to STR analysis (1) Accuracy of STR profiling using only cementum. (2) Cementum collection site. (3) Minimum amount of cementum required for STR profiling. STR: short tandem repeat; PCR: polymerase chain reaction.

For these samples, only teeth with no visually observed caries were used. Fresh teeth were randomly selected without considering gender or age. In contrast, all stale teeth samples were from men, as they were recovered from remains believed to be those of World War II combatants. Therefore, this study did not consider age or gender comparisons. The samples were anonymized after tooth extraction, and only samples with informed consent from the subject or family were used. This study was conducted at Tokyo Dental College between December 2021 and August 2023. The samples were cleaned with a neutral detergent and dried. Following this, the outer layer of the root was removed to prevent contamination from the periodontal ligament and calculus. During cementum collection, an Evans knife was used to prevent DNA damage due to frictional heat generated during cutting, ensuring that a layer of cementum was retained while excluding dentin [6,18,19]. Figure 2 shows a flowchart from sample preparation to DNA analysis.

**Figure 3:**
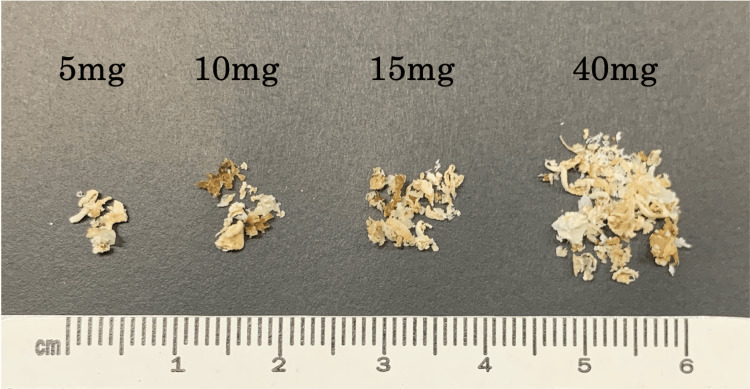
The four categories (5 mg, 10 mg, 15 mg, and 40 mg) of cementum Four categories (5, 10, 15, and 40 mg) of cementum were obtained from fresh and stale teeth.

First, to evaluate the accuracy of STR profiling using only the cementum, the STR type decision rates derived from DNA extracted from the cementum (40 mg) (Figure 3), termed method A, were compared to those from DNA extracted from the whole tooth after removing the cementum and pulverization, termed method B.

The STR type decision rate was calculated based on the number of loci with peaks ≥ 200 relative fluorescence units (RFU) detected, considering them as the type for each genetic locus out of 16 loci. The samples comprised 50 fresh teeth obtained from 50 individuals aged between 18 and 91 years (34 males and 16 females) and 50 stale teeth from 50 males of an undetermined age group.

Next, to determine the optimal site for cementum collection, we compared the STR type decision rates using cementum harvested from areas near the cervical line and the apex of the tooth root. The samples included 30 fresh teeth from 30 individuals aged 25-82 years (22 males and eight females) and 30 stale teeth from 30 males of unknown age. From these, 10 mg of cementum (Figure 3) was collected from each tooth in the areas near the cervical line and root apex.

Finally, to determine the minimum amount of cementum required for STR profiling, the quantity of cementum (5, 10, and 15 mg) was used (Figure 3), and the STR type decision rates for each category were compared. The samples consisted of 10 fresh teeth obtained from individuals aged 25-82 years (six males and four females) and 10 stale teeth from males of unknown age.

DNA purification and amplification

DNA extraction and purification were performed using the TBONE EX Kit (DNA Chip Research Inc., Tokyo, Japan) and the PureLink PCR Purification Kit (Thermo Fisher Scientific, Waltham, MA), producing a final elution volume of 50 μL. Subsequently, the DNA concentration (ng/μL) and purity ratio (absorbance at 260/280 nm) were measured using a NanoDrop spectrophotometer (Thermo Fisher Scientific, Waltham, MA). Each DNA concentration was diluted with TE buffer to achieve a concentration of 50 ng/μL. Fluorescent primers were amplified using the AmpFLSTR Identifiler Plus PCR Amplification Kit (Thermo Fisher Scientific, Waltham, MA). The polymerase chain reaction (PCR) amplification reaction mixture was prepared to a volume of 8.56 μL containing 3.16 μL of AmpFℓSTR Identifiler Plus Master Mix, 1.4 μL of AmpFℓSTR Identifiler Plus Primer Set, 0.5 μL of AmpliTaq Gold DNA Polymerase, and 175 ng of DNA. Thermal cycling conditions consisted of enzyme activation at 95°C for 11 minutes, followed by 34 cycles of denaturation at 94°C for 20 seconds, and annealing/extension at 59°C for three minutes. A final extension step was performed at 60°C for 10 minutes, followed by a final hold at 4°C if the PCR products were to remain in the thermal cycler for an extended time.

Sample electrophoresis and DNA analysis

PCR products were separated and detected using an Applied Biosystems Genetic Analyzer 3130 (Thermo Fisher Scientific, Waltham, MA); the run module was FragmentAnalysis36_POP7_1 and the dye set was G5, as described in the AmpFℓSTR Identifiler Plus Kit user guide [20]. For STR analysis, the AmpFLSTR Identifiler Plus PCR Amplification Kit (Thermo Fisher Scientific, Waltham, MA) was used to type 16 STR loci, including 15 autosomal markers (D21S11, CSF1PO, vWA, D8S1179, TH01, D18S51, D5S818, D16S539, D3S1358, D2S1338, TPOX, FGA, D7S820, D13S317, and D19S433) and the sex determination marker amelogenin. The assessment was conducted using the Gene Mapper Software v5 (Thermo Fisher Scientific, Waltham, MA).

Statistical analyses

Statistical analyses were performed using Student’s t-test, Tukey’s multiple comparison test, Pearson product-moment correlation coefficient, and Spearman's rank correlation coefficient. These tests were conducted with two-tailed significance levels of 0.05 and 0.01. The results are expressed as the mean ± standard deviation.

Ethics approval

The study protocol complied with the codes of ethical practice of the Tokyo Dental College. All study procedures and the use of human samples were approved by the Medical and Ethical Committee of the Tokyo Dental College (approval number: 1055).

## Results

Evaluation of the accuracy of STR profiling using only cementum

**Figure 4:**
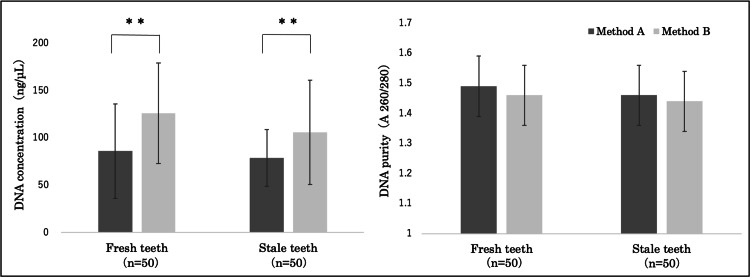
Comparison of DNA concentration and purity between methods A and B For both fresh and stale teeth, method A resulted in a significantly lower DNA concentration than method B (**p < 0.01); however, no significant difference in DNA purity was observed. Method A: DNA extracted from the cementum (40 mg). Method B: DNA extracted from the whole tooth after removing the cementum and pulverization.

A comparison was made between the concentration and purity of DNA derived from two extraction methods: method A, which involved extracting DNA from 40 mg of harvested cementum, and method B, which involved grinding the entire tooth after removing the cementum to extract DNA. The results showed that, in fresh teeth, the average DNA concentration for method A was 86±50 ng/μL, and the average DNA purity was 1.49±0.1. In contrast, the average DNA concentration for method B was 126±53 ng/μL, and the average DNA purity was 1.46±0.1. In stale teeth, the average DNA concentration for method A was 79±30 ng/μL, and the average DNA purity was 1.46±0.1. Conversely, the average DNA concentration for method B was 106±55 ng/μL, and the average DNA purity was 1.44±0.1. For both fresh and stale teeth, method A resulted in a significantly lower DNA concentration than method B (**p < 0.01). However, no significant differences were observed in DNA purity (Figure 4).

**Figure 5:**
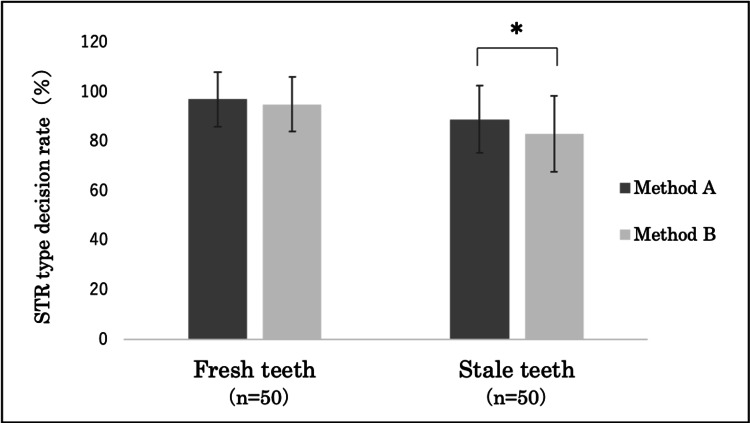
Comparison of STR type decision rates between methods A and B No significant differences were found between methods A and B for fresh teeth; however, a significantly higher STR type decision rate for stale teeth was observed for method A (*p < 0.05). Method A: DNA extracted from the cementum (40 mg). Method B: DNA extracted from the whole tooth after removing the cementum and pulverization. STR: short tandem repeat.

Additionally, the average STR typing decision rate for fresh teeth using method A was 97±11% (average: 15.5 of 16 loci), while for method B, it was 95±11% (average: 15.1 of 16 loci). In contrast, the average STR typing decision rate for stale teeth using method A was 89±14% (average: 14.2 of 16 loci), while for method B, it was 83±15% (average: 13.2 of 16 loci). For fresh teeth, no significant difference was observed between methods A and B; however, for stale teeth, a significantly higher typing decision rate was observed for method A than method B (*p < 0.05) (Figure 5).

**Figure 6:**
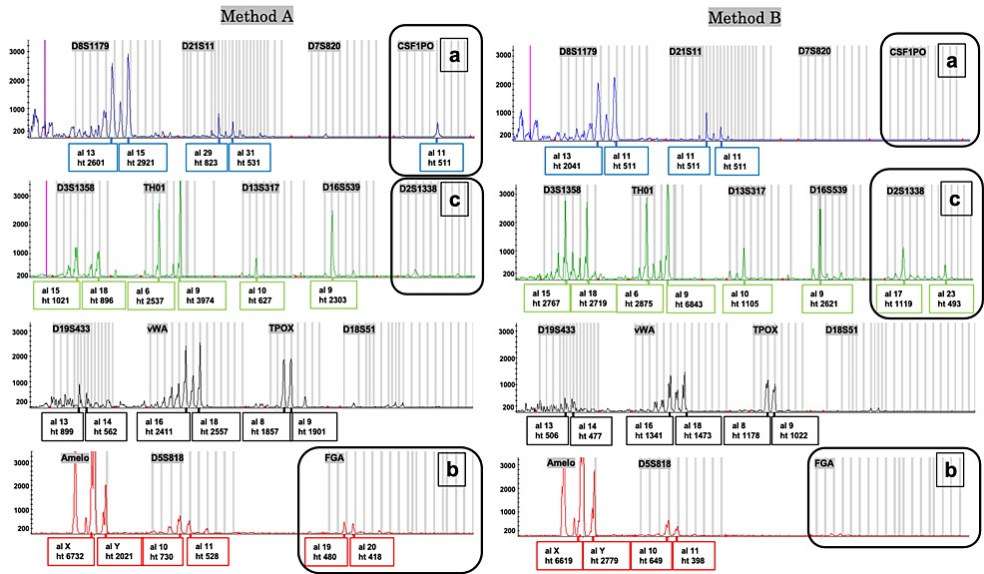
Examples of electropherograms depicting results for stale teeth following method A and method B For the CSF1PO (a) and FGA (b) loci, peaks were detected using method A, and profiling was possible; however, no peaks were detected using method B, indicating an allele dropout. In contrast, for the D2S1338 (c) locus, a peak was detected using method B, but no peak was detected using method A. Method A: DNA extracted from the cementum (40 mg). Method B: DNA extracted from the whole tooth after removing the cementum and pulverization.

An example of each electropherogram for methods A and B for stale teeth is shown in Figure 6.

For the CSF1PO and FGA loci, peaks were detected using method A, and profiling was possible; however, no peaks were detected using method B, indicating an allele dropout. Conversely, for the D2S1338 locus, a peak was detected using method B, but no peak was detected using method A. Overall, in fresh teeth, the proportion of peaks detected by both methods A and B was 93% (742 of 800 loci). The proportion of peaks detected by method A but not by method B was 4% (32 of 800 loci), while the proportion of peaks detected by method B but not by method A was 1% (10 of 800 loci), and the proportion of peaks not detected by either method A or method B was 2% (16 of 800 loci). In contrast, for the stale teeth, 76% (611 of 800 loci) of the peaks were detected by both methods A and B. Furthermore, 12% (94 of 800 loci) of the peaks were detected by method A but not by method B, while 7% (54 of 800 loci) of the peaks were detected by method B but not by method A, and 5% (41 of 800 loci) of the peaks were not detected by either method A or B.

Evaluation of the cementum collection site

**Figure 7:**
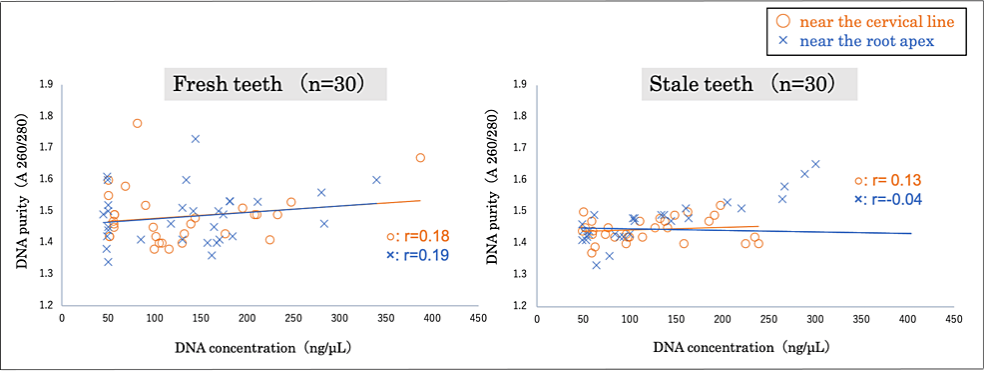
Correlation between the concentration and purity of DNA in cementum obtained from the areas near the cervical line and root apex No correlation was found between DNA concentration and purity in either fresh or stale tooth samples. Statistical analysis was performed using the Pearson product-moment correlation coefficient.

After comparing the average concentration and purity of DNA extracted from 10 mg of cementum in the areas near the cervical line and the root apex of fresh teeth, it was found that the average DNA concentration in the area near the cervical line was 125±79 ng/μL, and the DNA purity was 1.5±0.1. Meanwhile, the average DNA concentration in the area near the root apex was 133±78 ng/μL, and the DNA purity was also 1.5±0.1. In contrast, in stale teeth, it was found that the average DNA concentration in the area near the cervical line was 119±58 ng/μL, and the DNA purity was 1.5±0.0. Moreover, the average DNA concentration in the area near the root apex was 139±94 ng/μL, and the DNA purity was also 1.4±0.1. Correspondingly, no significant differences in DNA concentration and purity were observed between the areas near the cervical line and the root apex in fresh or stale teeth. Furthermore, no correlation was found between DNA concentration and purity in either fresh or stale tooth samples. While some DNA samples exhibited high concentrations despite low purity, all samples demonstrated a minimum DNA concentration of 50 ng/μL and a DNA purity of at least 1.3 (Figure 7).

**Figure 8:**
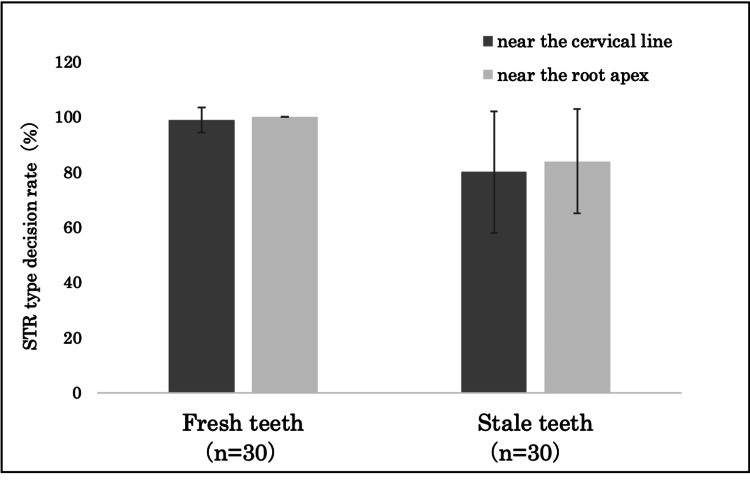
Comparison of STR type decision rates for cementum from the area near the cervical line and root apex There were no significant differences in STR type decision rates for fresh and stale teeth. Statistical analysis was performed using the Student’s t-test. STR: short tandem repeat.

Furthermore, in fresh teeth, the STR type decision rate was 99±4.6% (average: 15.9 of 16 loci) for the area near the cervical line and 100% (16 out of 16 loci) for the area near the root apex. In contrast, for stale teeth, the STR type decision rate was 80±22% (average: 12.8 of 16 loci) for the area near the cervical line and 84±19% (average: 13.4 of 16 loci) for the area near the root apex. No significant difference in STR type decision rates was observed between the areas near the cervical line and the root apex in both fresh and stale teeth (Figure 8).

Determination of the minimum amount of cementum required for STR profiling

**Figure 9:**
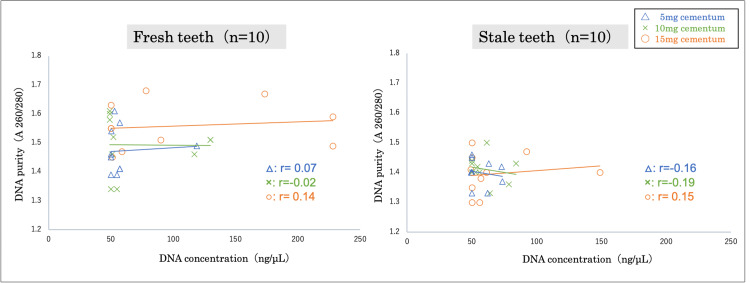
Correlation between DNA concentration and purity in 5 mg, 10 mg, and 15 mg of cementum No correlation between DNA concentration and purity was observed in either fresh or stale tooth samples. Statistical analysis was performed using Spearman's rank correlation coefficient.

The amount of cementum was classified into three categories (5, 10, and 15 mg), and the concentration and purity of the extracted DNA were compared. The average DNA concentration in fresh teeth was 60±21 ng/μL for 5 mg cementum, 73±36 ng/μL for 10 mg cementum, and 106±75 ng/μL for 15 mg cementum. The average DNA purity of fresh teeth was 1.5±0.1 across all quantities of cementum (5 mg, 10 mg, and 15 mg). The average DNA concentration of stale teeth was 57±10 ng/μL for 5 mg cementum, 60±12 ng/μL for 10 mg cementum, and 66±32 ng/μL for 15 mg cementum. Moreover, the average DNA purity of stale teeth was 1.4±0.1 across all quantities of cementum (5, 10, and 15 mg). Furthermore, no correlation between DNA concentration and purity was observed in either fresh or stale tooth samples. However, it was shown that the minimum DNA concentration and purity were at least 50 ng/μL and 1.3, respectively, across all samples for 5 mg, 10 mg, and 15 mg of cementum (Figure 9).

**Figure 10:**
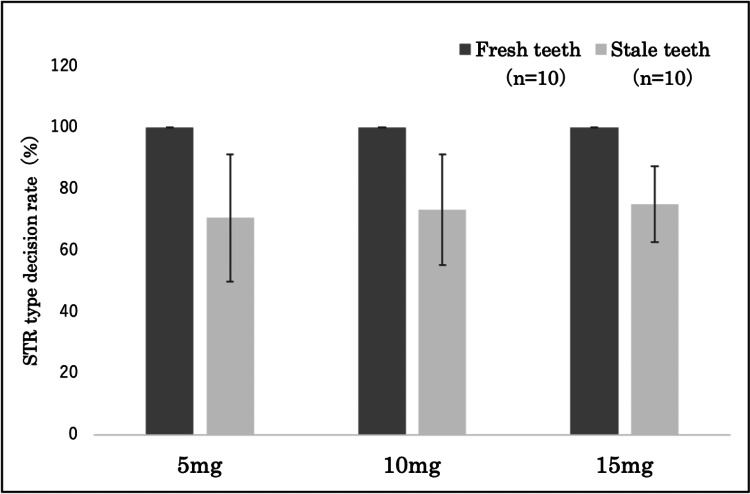
Comparison between STR type determination rates for 5 mg, 10 mg, and 15 mg of cementum The fresh tooth samples exhibited STR type decision rates of 100% across all amounts of cementum (5 mg, 10 mg, and 15 mg). For stale teeth, there were no significant differences in STR type decision rates across all amounts of cementum (5 mg, 10 mg, and 15 mg). Statistical analysis was performed using Tukey’s multiple comparison test. STR: short tandem repeat.

Additionally, when comparing the STR type decision rates, fresh teeth exhibited a 100% decision rate (16 of 16 loci) across all samples for 5, 10, and 15 mg of cementum. On the contrary, stale teeth exhibited STR type decision rates of 71±21% (average: 11.3 of 16 loci) for 5 mg cementum, 73±18% (average: 11.7 of 16 loci) for 10 mg cementum, and 75±12% (average: 12.4 of 16 loci) for 15 mg cementum; no significant difference was observed between each cementum amount (Figure 10).

**Figure 11:**
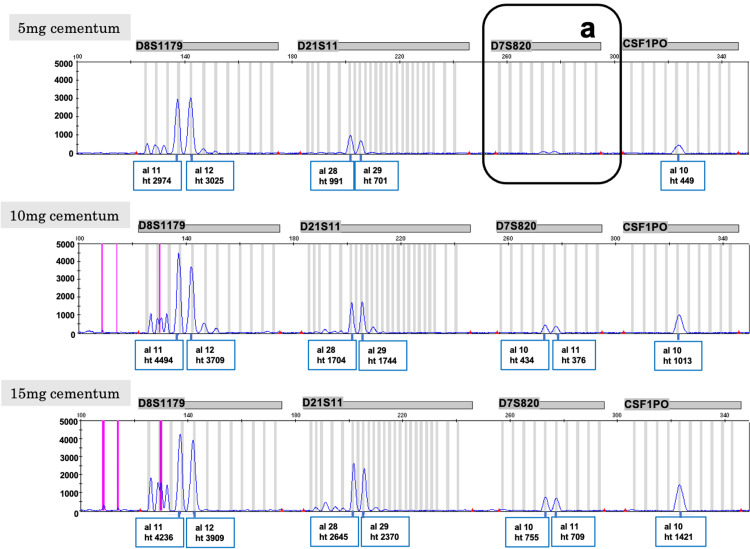
Electropherograms of 5 mg, 10 mg, and 15 mg cementum A notable occurrence of allele dropout (a) was observed in 5 mg of cementum from stale teeth, with a trend indicating increased peak height concomitant with an increase in cementum quantity.

However, a notable occurrence of allele dropout was observed in 5 mg of cementum from stable teeth, with a trend indicating increased peak heights concomitant with an increase in cementum quantity (Figure 11).

## Discussion

In this study, we investigated the utility of STR profiling using only the cementum, the optimal site for cementum collection, and the minimum amount of cementum required for STR profiling. The results demonstrated that the profiling accuracy of DNA extracted from cementum was comparable to that of DNA from dental pulp and dentin. Furthermore, the collection of cementum from near the cervical line or from the root apex areas did not show significant differences in DNA profiling accuracy, indicating that securing at least 5 mg of cementum was sufficient to ensure precise DNA profiling. These findings suggest that DNA profiling using only cementum is viable even in teeth that have been subjected to a long postmortem interval.

DNA polymorphisms are classified into categories based on the number of repeating base units, such as STR and variable number of tandem repeats (VNTR). In particular, STR profiling using commercially available kits is extensively used not only for blood relationship identification but also for the personal identification of unidentified bodies, with databases of STR types established in various countries worldwide [21,22]. However, the accuracy of identification may be insufficient with only STR profiling. Performing additional forensic examinations allows for more precise individual identification. Recent studies have used stable isotopes from bones and nails to estimate diet and natal origins. Further application of these methods enables the estimation of birth regions and racial differentiation through the analysis of stable isotope ratios in dental enamel [23,24]. Additionally, it has been reported that age can be estimated by determining the racemization ratio using amino acids in dentin [25]. As these analyses can be performed using only a part of a tooth, it is feasible to conduct personal identification through DNA typing in conjunction with other forensic examinations by developing a DNA profiling method using only cementum, making it highly valuable. Cementum contains the requisite DNA concentration for DNA profiling, and the feasibility of STR profiling using cementum alone has been reported. However, these reports were based on teeth sampled within three years postmortem, and there have been no investigations using teeth recovered from bodies after a long postmortem period or teeth that have been stored under poor conditions [11-16]. DNA is susceptible to degradation over time postmortem and owing to the storage conditions of the samples, resulting in a decrease in DNA yield and purity [26,27]. Specifically, DNA present in teeth that have been left outdoors is particularly prone to degradation due to temperature, humidity, bacteria, and ultraviolet radiation, and it has been reported that abandonment in soil can induce inhibitory effects on PCR [8]. However, even in teeth where DNA degradation was observed, it was demonstrated that DNA extracted from cementum had equivalent genotyping accuracy to DNA extracted from dental pulp or dentin. Cementum possesses numerous small cavities and is isolated from the external environment, rendering it less susceptible to damage from physical forces and bacterial contamination [28]. Based on these results, it is suggested that DNA identification using the cementum alone is viable, even in teeth that have been exposed to an extended postmortem period. Furthermore, this study examined the impact of sampling sites within the cementum on profiling accuracy. Recently, reports have compared DNA concentrations in two areas of the entire tooth root, specifically near the cervical line and the root apex. It has been indicated that the DNA concentration is significantly higher near the root apex than near the cervical line [17]. However, regardless of whether the samples were fresh or stale, the current study did not observe a significant difference in DNA concentration or profiling accuracy between the areas near the cervical line and the root apex. These conflicting results may be attributed to individual variations resulting from factors such as the number of cementocytes or type of tooth [29,30]. Based on the above results, it is suggested that while the distribution of cementocytes may cause variations in DNA concentration when cementum is harvested from areas near the cervical line and root apex, it does not affect the accuracy of STR profiling. In the evaluation of the minimum amount of cementum required for identification, it was demonstrated that a 100% STR type decision rate was achieved using 5 mg of cementum from teeth extracted within six months, indicating high determination accuracy. In contrast, in teeth that had been exposed to an extended postmortem period, the STR type decision rate was 71±21% for 5 mg cementum, 73±18% for 10 mg cementum, and 75±12% for 15 mg cementum, revealing no significant differences among these determination rates. However, for 40 mg cementum, the STR type decision rate was 89±14%, indicating that while STR profiling is possible with as little as 5 mg cementum, obtaining as much cementum as feasible is recommended for optimal results.

Lastly, this study focused solely on STR typing to assess the utility of cementum alone for DNA profiling. However, in DNA profiling, STR profiling is frequently used in combination with single nucleotide polymorphism analysis using mitochondrial DNA. Therefore, further investigations are required to determine the feasibility of using cementum alone in other DNA identification methods. In addition, in this study, teeth with visually confirmed caries in the tooth roots were excluded from the samples; as such, the impact of caries on the analysis results was not determined. Furthermore, since the samples in this study used cementum from teeth obtained from people over 18 years of age, it is necessary to examine whether similar results can be obtained for the teeth of younger people with less cementum formation.

## Conclusions

DNA profiling using only cementum is valuable not only for teeth extracted within six months but also for teeth exposed to extended postmortem intervals. In other words, this method allows for DNA profiling without pulverizing the tooth, even in samples where DNA degradation may have occurred. Furthermore, because it is possible to preserve the original structure of the tooth after identification, it is expected that this method can be used in conjunction with other forensic examinations, or for the re-evaluation of DNA profiling.
